# Intravascular Large B-Cell Lymphoma in Acute Hypoxic Respiratory Failure

**DOI:** 10.1155/2023/9192396

**Published:** 2023-08-12

**Authors:** Kei Yanagisawa, Akiko Kameyama, Hiroshi Miyama, Koutarou Mori, Masao Hattori, Hiroshi Imamura, Kenichi Nitta

**Affiliations:** Department of Emergency and Critical Care Medicine, Shinshu University School of Medicine, 3-1-1 Asahi, Matsumoto, Nagano 390-8621, Japan

## Abstract

Intravascular large B-cell lymphoma, an extranodal large B-cell lymphoma, is a rare hematological malignancy with only a few reports of lung involvement. We report a case of intravascular large B-cell lymphoma with acute hypoxic respiratory failure and interstitial lung disease diagnosed via random skin biopsies. A 54-year-old woman presented with fever, cough, and dyspnea. Computed tomography imaging revealed findings concerning interstitial lung disease. The patient's respiratory status worsened despite the treatment with antibiotics and steroids. Generalized edema and thrombocytopenia also developed. Intravascular large B-cell lymphoma was clinically suspected and ultimately diagnosed by skin biopsy, although she had no apparent skin lesions. The patient's condition considerably improved after chemotherapy. Intravascular large B-cell lymphoma should be considered in patients with acute respiratory failure and interstitial lung lesions.

## 1. Introduction

Intravascular large B-cell lymphoma (IVLBCL) is a rare extranodal B-cell lymphoma with selective proliferation in the microvasculature [1]. IVLBCL does not involve lymphadenopathy, common in malignant lymphoma, and exhibits nonspecific symptoms, including fever, general malaise, dementia, and dyspnea. Therefore, its diagnosis is often difficult. Furthermore, primary lung involvement is rare and poorly described [2].

This paper reports a case of IVLBCL with acute hypoxic respiratory failure and interstitial lung disease diagnosed using random skin biopsies.

## 2. Case Presentation

A 54-year-old woman with dyspnea presented to a community hospital with a fever and cough for a week. Chest computed tomography (CT) showed bilateral ground-glass opacities (GGOs) in the entire lung field, and the patient was referred to our hospital. She had no prior medical history besides moderate smoking between the ages of 20 and 25 years. Upon examination, she had the following: a body temperature of 37.6°C, blood pressure of 80/52 mmHg, heart rate of 90 beats/min, respiratory rate of 18 breaths/min, oxygen saturation (SpO_2_) of 94% at 3 L/min of oxygen via a nasal prong, normal heart sounds, diffuse fine crackles on chest auscultation, and 1+ pitting edema noted in her feet as well as dorsal surfaces, without any superficial lymph nodes or neurological abnormalities.

**Figure 1:**
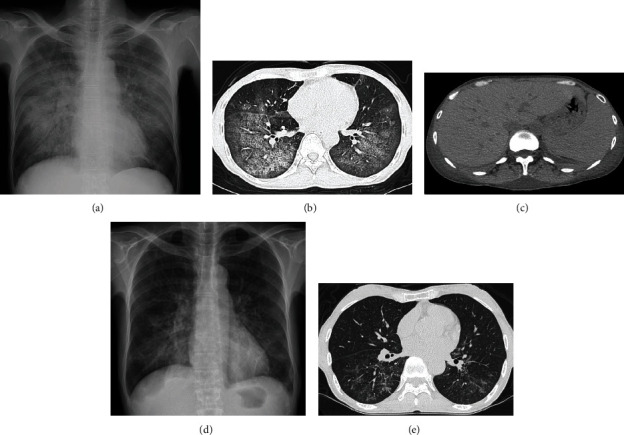
Chest radiography and computed tomography (CT) during hospitalization. (a) Chest radiography on admission showing ground-glass opacity in bilateral lung fields. (b) Chest CT on admission showing bilateral diffuse, hazy ground-glass infiltrates throughout the lungs. (c) Abdominal CT on admission showing mild hepatosplenomegaly. (d) Chest radiography on day 20 showing marked improvement in lung shadowing. (e) Chest CT on day 27 showing marked improvement in lung shadowing.

**Figure 2:**
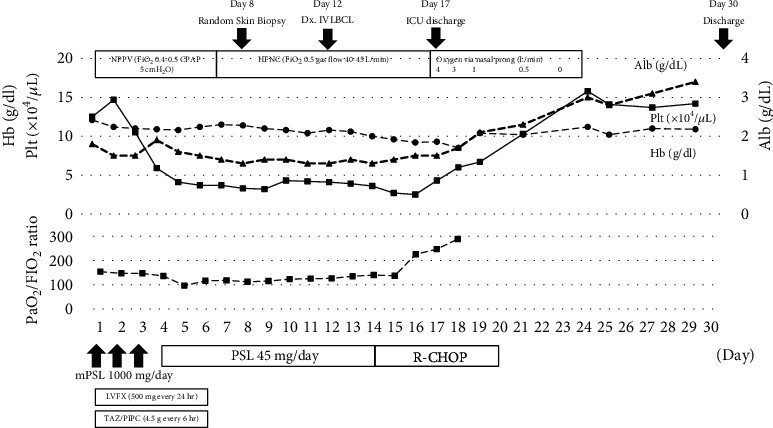
Time course of treatment of intravascular large B-cell lymphoma. NPPV: noninvasive positive pressure ventilation; HFNC: high-flow nasal cannula; Alb: albumin; Hb: hemoglobin; Plt: platelet; mPSL: methylprednisolone; PSL: prednisolone; LVFX: levofloxacin; TAZ/PIPC: tazobactam/piperacillin; R-CHOP: rituximab, cyclophosphamide, doxorubicin, vincristine, and prednisone. The patient's respiratory condition did not improve after administering antibiotics or high steroid doses. NPPV and HFNC were used for respiratory support. Blood tests revealed a further decline in platelet count, albumin, and hemoglobin levels. On day 8, a random skin biopsy was performed; on day 12, the patient was diagnosed with IVLBCL after a skin biopsy. On day 14, the patient received R-CHOP therapy. After one chemotherapy cycle, the patient's dyspnea gradually improved, and oxygen therapy was discontinued on day 24. The patient was discharged on day 30.

**Figure 3:**
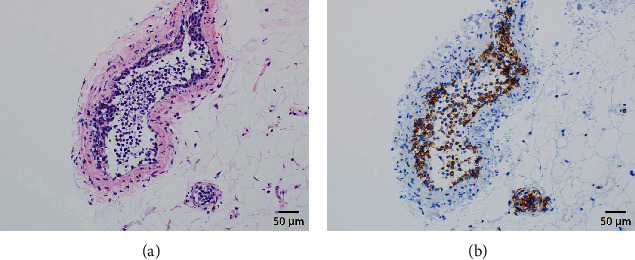
Hematoxylin-eosin (H&E) staining and CD20 immunostaining of intravascular B lymphocytes from the thigh skin biopsies (400x). (a) Atypical cells are packed in abnormal vessels (H&E staining, 20x). (b) Immunohistochemistry of atypical CD20-positive cells (20x).

The laboratory test results were 12.1 g/dL hemoglobin (reference range, 11.6-14.8), 749 IU/L lactate dehydrogenase (LDH) (124-222), 1.8 g/dL albumin (4.1-5.1), and a platelet count of 12.7 × 10^4^/*μ*L (15.8-34.8), while SARS-CoV-2 tests (PCR and antibody) were negative. Chest radiography and CT showed diffuse interstitial lung shadows (Figures 1(a) and 1(b)), while abdominal CT revealed mild hepatosplenomegaly (Figure 1(c)). Therefore, the following were suspected: idiopathic or collagen vascular disease-associated interstitial pneumonitis, alveolar hemorrhage syndrome, viral or bacterial pneumonia, or hypersensitivity pneumonitis. Treatment was initiated with methylprednisolone (1000 mg/day) for 3 days, followed by tazobactam piperacillin (18 g/day) and levofloxacin (500 mg/day) for the next 6 days, providing coverage for atypical pathogens (Figure 2). However, the patient's respiratory status worsened despite the treatment with antibiotics and steroids. She was placed on noninvasive ventilatory support or high-flow nasal oxygen for hypoxemia. The fraction of inspired oxygen was regularly adjusted to maintain SpO_2_ above 92%. Blood tests showed a further decline in platelet count (5.9 × 10^4^/*μ*L) and hemoglobin level (10.8 g/dL) on day 4. At the same time, her generalized edema gradually worsened despite the use of diuretics. The screening results were negative for antinuclear antibodies, autoantibodies, and several infectious diseases. Based on thrombocytopenia and hepatosplenomegaly, we suspected hematologic disease; therefore, we measured soluble interleukin 2 receptor (sIL-2R) levels on day 7. The sIL-2R levels were elevated (9465 U/mL [121-613]). IVLBCL was suspected because of hepatosplenomegaly, thrombocytopenia, diffuse lung disease, and elevated sIL-2R levels. Random skin and bone marrow biopsies were performed on day 8, and thigh skin biopsies sampled on day 12 revealed large, atypical lymphoma cells within the capillaries (Figure 3(a)) positive for CD20 (Figure 3(b)) but negative for CD3, CD5, and CD10. Small clusters of CD20-positive cells were found in the bone marrow without hemophagocytosis.

Consequently, the patient was diagnosed with IVLBCL. She received rituximab, cyclophosphamide, doxorubicin, vincristine, and prednisone on day 14. After one chemotherapy cycle, her dyspnea and generalized swelling rapidly improved, and oxygen therapy was discontinued on day 24. The chest shadows improved dramatically on radiography on day 20 (Figure 1(d)) and on CT on day 27 (Figure 1(e)). The patient received six chemotherapy cycles after discharge on day 30. Currently, she is under careful observation with no recurrence signs.

## 3. Discussion

We report a case of IVLBCL with acute hypoxic respiratory failure and interstitial lung disease. The extrapulmonary findings of splenomegaly, thrombocytopenia, and high sIL-2R and LDH levels were suggestive of IVLBCL. After a straightforward skin biopsy and specific treatment, the patient recovered.

IVLBCL is a distinct type of extranodal diffuse large B-cell lymphoma and affects patients between 34 and 90 years, with a median age range between 60 and 70 years [3]. In Japan, an estimated 300–400 patients annually develop IVLBCL [4]. The main characteristic of IVLBCL is the selective lymphoma proliferation within the small blood vessel lumen [1]. Its clinical symptoms vary widely and are nonspecific, making diagnosis difficult. In a previous retrospective Japanese cohort study, the most common clinical symptoms in IVLBCL were fever, general malaise, neurological symptoms, and dyspnea, observed in 73%, 25%, 25%, and 19% of patients, respectively [5]. Meanwhile, skin eruption was observed in 6% of patients [5]. Despite the absence of lymphadenopathy, various organ enlargements, such as the liver, spleen, kidney, and adrenal gland, were observed as common abnormalities in IVLBCL on imaging. Hepatosplenomegaly was observed in 50%–80% of patients [5, 6].

Although the abdominal CT on admission showed splenomegaly, we considered it nonspecific. Anemia, thrombocytopenia, hypoalbuminemia, and high levels of serum LDH and sIL-2R are frequently identified by laboratory investigations [5, 6], as in our case. However, on admission, the results of the laboratory tests were normal, except for LDH levels and hypoalbuminemia.

Respiratory symptoms in IVLBCL usually accompany lung involvement, including coughing, sputum production, and dyspnea. The lung appearance on chest CT varies: interlobular septal thickening, centrilobular nodules, and GGOs. Matsue et al. analyzed the clinical data of 42 patients with IVLBCL over the past 20 years and identified the presence of pleural effusion in 15/42 (40.0%) patients and GGOs in 9/42 (21.4%) patients via chest CT [5]. The GGO area on CT images might histopathologically correlate with those of the thickened alveolar septa and perivascular spaces because of the small vessels distension, such as septal capillaries, venules, and arterioles filled with atypical lymphoid cells [6].

In this case, the patient was diagnosed with IVLBCL via a random skin biopsy because lung biopsies are invasive and can exacerbate the respiratory condition. Random skin biopsy is now a standard diagnostic procedure for patients with suspected IVLBCL in Japan but not in Western countries [7]. Enzan et al. reported that IVLBCL lesions were rare in the dermis but plenty in the hypodermic adipose tissues [8]. In Western countries, punch biopsies might have limited diagnostic capability in IVLBCL because of insufficient amounts of hypodermal adipose tissue sampled [7]. Therefore, if IVLBCL is suspected in a patient with hypoxemia and no skin lesions are present, random skin biopsies plus immunohistochemical staining for CD20 and CD34 can reduce the diagnosis and therapy times and are less invasive.

## 4. Conclusion

Physicians must consider IVLBCL in patients with interstitial lung lesions, hepatosplenomegaly, and high LDH levels as a differential diagnosis for acute respiratory failure, although it is a rare disease.
